# Endoscopic Controlled Radial Expansion Balloon Dilatation in Infantile Achalasia: A Case Series of Four Infants

**DOI:** 10.7759/cureus.97207

**Published:** 2025-11-19

**Authors:** Devendra S Kushwaha, Arghya Samanta, Gautam Ray, Biswadeep Roy

**Affiliations:** 1 Department of Gastroenterology, School of Digestive and Liver Diseases, Institute of Post Graduate Medical Education and Research and Seth Sukhlal Karnani Memorial Hospital, Kolkata, IND; 2 Department of Pediatric Gastroenterology, School of Digestive and Liver Diseases, Institute of Post Graduate Medical Education and Research and Seth Sukhlal Karnani Memorial Hospital, Kolkata, IND

**Keywords:** controlled radial expansion (cre) balloon dilation, endoscopic dilatation, failure to thrive, gastroesophageal junction (gej), infantile achalasia

## Abstract

Achalasia cardia is an uncommon esophageal motility disorder that is often misdiagnosed as gastroesophageal reflux disease (GERD). Although rare in infancy, it presents unique diagnostic and therapeutic challenges compared to adults. We describe four infants who presented with early-onset vomiting and feeding difficulties and were diagnosed with achalasia cardia based on characteristic findings on timed barium esophagogram (TBE). None of our patients had features of Allgrove (Triple-A) syndrome, which is characterized by achalasia, alacrimia, and adrenal insufficiency. Serial controlled radial expansion (CRE) balloon dilatation was performed under direct endoscopic vision to relieve lower esophageal sphincter (LES) obstruction. The procedure was uneventful, and three (75%) infants showed marked clinical improvement, remaining symptom-free at a median follow-up of 4.5 months. Congenital esophageal stenosis, an important differential diagnosis in this age group, was carefully excluded based on characteristic endoscopic and radiologic findings. Infantile achalasia is diagnostically challenging because of symptom overlap with GERD and difficulty in performing manometry, limited cooperation for functional studies, and the requirement for specialized pediatric endoscopic equipment due to the small caliber of the esophagus. Timed barium esophagogram remains the most useful diagnostic tool, while CRE balloon dilatation provides a safe and minimally invasive alternative to surgery. Endoscopic CRE balloon dilatation appears to be an effective treatment option for infantile achalasia, with sustained symptom remission and nutritional recovery highlighting its feasibility and clinical value.

## Introduction

Achalasia cardia is a primary esophageal motility disorder characterized by the failure of the lower esophageal sphincter (LES) to relax during swallowing and the loss of coordinated peristalsis of the esophageal body. The condition is rarely reported in children and is exceedingly uncommon in infants [1]. Its symptoms often mimic gastroesophageal reflux disease (GERD) or other causes of feeding difficulty, leading to delayed diagnosis. Congenital esophageal stenosis is an important structural differential diagnosis in infants with vomiting and feeding difficulties; unlike achalasia, it typically shows a fixed, focal narrowing on radiologic imaging and a nonnegotiable, anatomically constricted segment on endoscopy, making careful radiologic and endoscopic distinction essential [2]. Esophageal manometry remains the diagnostic gold standard, yet it is technically challenging in infants because of difficulty in catheter placement, movement, or crying artifacts that distort the tracings, and the limited availability of modified pediatric protocols and specialized equipment; hence, a timed barium esophagogram (TBE) often provides the key diagnostic clue. Various therapeutic modalities have been described, including pharmacologic therapy (calcium channel blockers and nitrates), botulinum toxin injection, pneumatic dilation, and Heller’s myotomy, each with limitations in the pediatric population. Herein, we report four infants with achalasia cardia managed with controlled radial expansion (CRE) balloon dilatation. Controlled radial expansion (CRE) balloon dilatation, performed under direct endoscopic vision, offers a graded, minimally invasive alternative.

## Case presentation

Case 1

**Figure 1:**
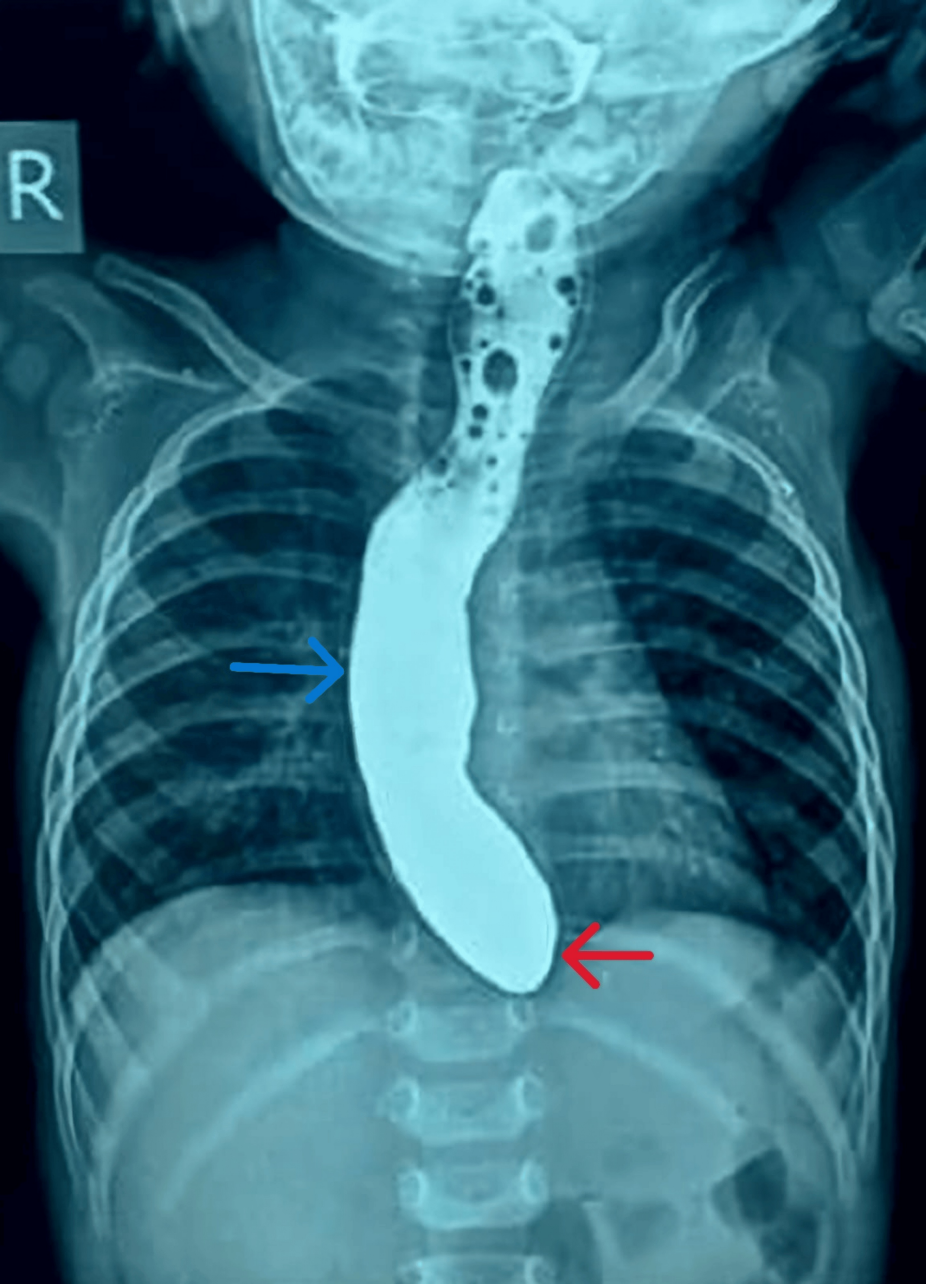
Timed barium esophagogram showing a dilated esophagus (blue arrow) with tapering at the gastroesophageal junction (red arrow), producing the classical “bird-beak” appearance characteristic of achalasia cardia.

A four-month-old girl presented with recurrent non-bilious vomiting, repeated chest infections, and failure to thrive. Her body weight and length were 3.7 kg (z-score of less than -3 standard deviation {SD}) and 55 cm (z-score of less than -3 SD), respectively. She had received proton-pump inhibitors (PPIs) for six weeks without improvement. The baseline demographic and clinical characteristics are summarized in Table 1. The timed barium esophagogram (TBE) demonstrated a markedly dilated thoracic esophagus with tapering at the gastroesophageal junction, producing the classical “bird-beak” appearance, as shown in Figure 1.

**Table 1:** The baseline demographic and clinical characteristics of all four infants. SD: standard deviation

Parameter	Case 1	Case 2	Case 3	Case 4
Age (months)	4	9	6	10
Sex	Girl	Boy	Girl	Boy
Presenting complaints	Recurrent non-bilious vomiting; recurrent chest infections	Recurrent aspirations; non-bilious vomiting	Regurgitation of uncurdled milk	Dysphagia
Failure to thrive	Yes	Yes	Yes	Yes
Weight for age (z-score)	Less than -3 SD	-2.4 SD	-2.9 SD	-2.2 SD
Length for age (z-score)	Less than -3 SD	-2.6 SD	-2.3 SD	-2.3 SD
Alacrimia/hyperpigmentation	Absent	Absent	Absent	Absent

**Figure 2:**
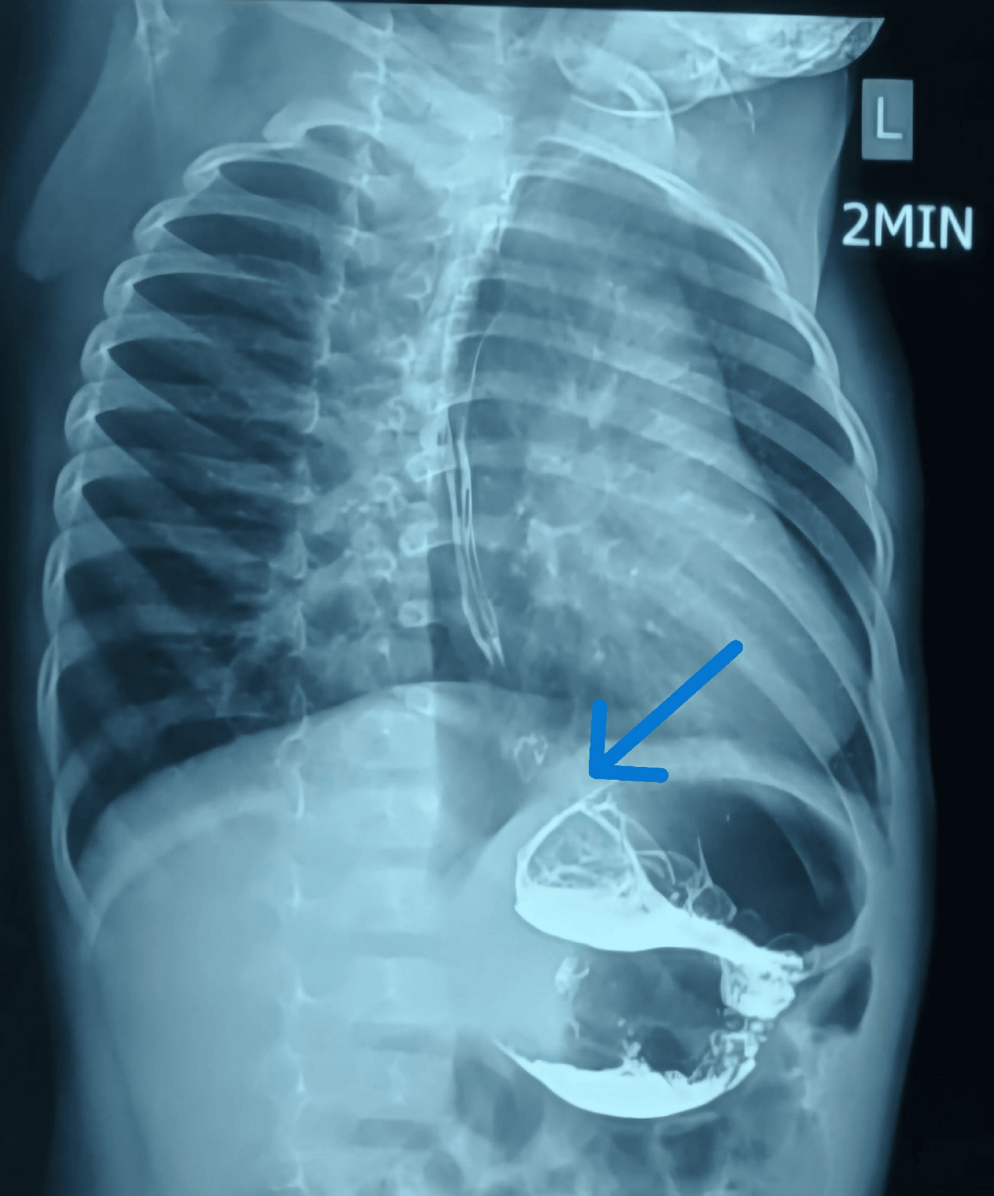
Post-controlled radial expansion (CRE) balloon dilatation timed barium esophagogram showing improved esophageal emptying at two minutes.

Upper gastrointestinal endoscopy (UGIE) confirmed a dilated esophagus and a tight but negotiable gastroesophageal junction. Esophageal manometry could not be performed because of technical constraints. Serial endoscopic controlled radial expansion (CRE) balloon dilatations were carried out using 10 mm, 12 mm, and 15 mm balloons, each inflated for three minutes under direct endoscopic vision. The child showed prompt symptomatic relief, and repeat TBE revealed a substantial reduction in the barium column height, as shown in Figure 2. After six months of follow-up, she remained asymptomatic, with her body weight being 6.6 kg (z-score of -2.2 SD). Diagnostic findings, procedure details, and outcomes are summarized in Table 2.

**Table 2:** Diagnostic findings, intervention details, and outcomes. TBE, timed barium esophagogram; UGIE, upper gastrointestinal endoscopy; GEJ, gastroesophageal junction; CRE, controlled radial expansion; HM, Heller’s myotomy

Parameters	Case 1	Case 2	Case 3	Case 4
TBE (type of achalasia)	Dilated esophagus with narrowing at GEJ (straight type)	Dilated esophagus with narrowing at GEJ (straight type)	Dilated esophagus with narrowing at GEJ (straight type)	Dilated esophagus with narrowing at GEJ (straight type)
UGIE	Dilated esophagus, pent-up secretion, tight GEJ, and crossed with difficulty	Dilated esophagus, tight GEJ with retained food residue, and crossed with difficulty	Dilated esophagus, pent-up secretion, tight GEJ, and crossed with difficulty	Dilated esophagus, tight GEJ, and crossed with difficulty
Esophageal manometry	Not done	Not done	Not done	Not done
Number of CRE sessions (balloon size, mm)	3 (10→12→15)	3 (12→15→20)	3 (12→15→20)	4 (12→15→20→20)
Complications	None	None	None	None
Follow-up duration (months)	6	4	4.5	Lost to follow-up
Outcome	Asymptomatic; weight gain	Asymptomatic; weight gain	Asymptomatic; weight gain	Persistent dysphagia; referred for HM

Case 2

**Figure 3:**
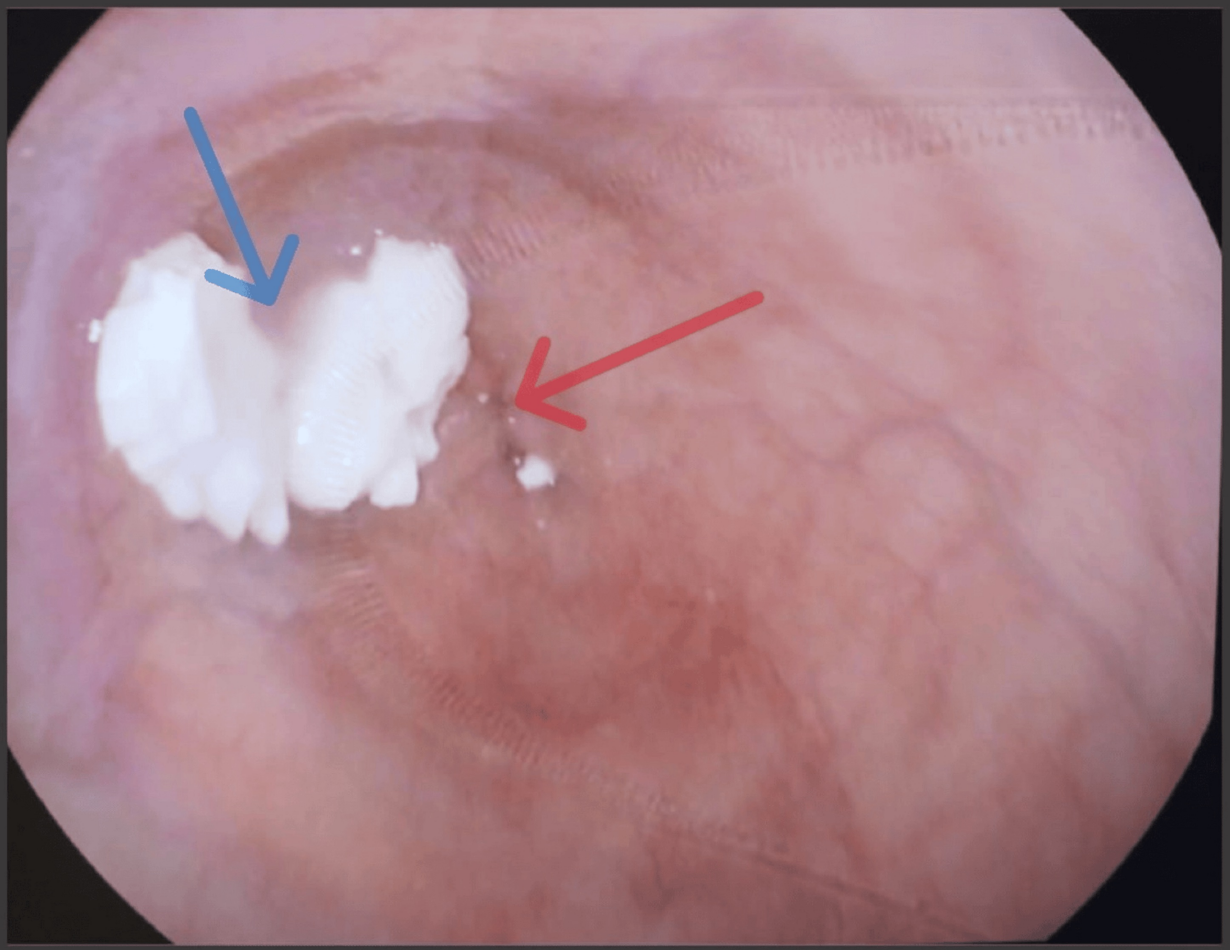
Upper gastrointestinal endoscopic image demonstrating food residue within a dilated esophagus (blue arrow) and a tight, non-relaxing gastroesophageal junction (red arrow) consistent with achalasia cardia.

A nine-month-old boy presented with recurrent aspirations and non-bilious vomiting of uncurdled milk since the age of five months. His weight and length were 6.7 kg (z-score of -2.4 SD) and 62 cm (z-score of -2.6 SD), respectively. The baseline demographic and clinical characteristics are summarized in Table 1. TBE showed a typical “bird-beak” sign, and UGIE confirmed a tight gastroesophageal junction with retained food residue in the distal esophagus, as shown in Figure 3.

CRE balloon dilatation was performed sequentially with 12 mm, 15 mm, and 20 mm balloons, each inflated for three minutes under direct endoscopic vision. The procedure was uneventful. Post procedure, his symptoms resolved, and follow-up imaging demonstrated improved esophageal emptying. After four months of follow-up, he remained symptom-free with appropriate weight gain. Diagnostic findings, procedure details, and outcomes are summarized in Table 2.

Case 3

A six-month-old girl presented with persistent regurgitation of uncurdled milk since two months of age. Her weight and length were 5.2 kg (z-score of -2.9) and 60.4 cm (z-score of -2.3), respectively. The baseline demographic and clinical characteristics are summarized in Table 1. TBE demonstrated a dilated esophagus with distal tapering consistent with achalasia. She underwent three serial CRE balloon dilatations with 12 mm, 15 mm, and 20 mm balloons, each inflated for three minutes under direct endoscopic vision, without any complications. She experienced rapid clinical improvement and remained asymptomatic throughout 4.5 months of follow-up. Diagnostic findings, procedure details, and outcomes are summarized in Table 2.

Case 4

A 10-month-old boy presented with gradually progressive dysphagia for both solid and liquid foods for the last four months. The baseline demographic and clinical characteristics are summarized in Table 1. He was diagnosed with achalasia cardia based on typical TBE findings. He underwent four sequential CRE balloon dilatations using 12 mm, 15 mm, and 20 mm balloons (20 mm repeated at the fourth session), each inflated for three minutes under direct endoscopic vision. Despite technically successful procedures, dysphagia persisted, and the patient was referred for Heller’s myotomy. He was subsequently lost to follow-up. Diagnostic findings, procedure details, and outcomes are summarized in Table 2.

The demographic, clinical characteristics, diagnostic findings, procedure details, and outcomes for all four infants are summarized in Table 1 and Table 2.

## Discussion

The reported incidence of achalasia cardia is 0.5-1 per 100,000 individuals, with 3%-5% of cases occurring in children and less than 1% in infants [1]. All four infants in our series initially received therapy for presumed GERD, resulting in delayed diagnosis. Although congenital esophageal stenosis is an important differential diagnosis in infants with feeding difficulties and esophageal dilatation, the radiologic pattern of smooth symmetric tapering at the gastroesophageal junction, normal endoscopic anatomy without anatomic narrowing, and the favorable clinical response to CRE balloon dilatation in our patients strongly supported the diagnosis of infantile achalasia. Careful radiologic and endoscopic evaluation remains essential to distinguish these two entities, as their management strategies and outcomes differ significantly [2]. Manometry, the diagnostic gold standard, typically demonstrates elevated LES resting pressure with incomplete LES relaxation during swallows [1,3]. However, it is often not feasible in infants because of technical limitations. Consequently, TBE remains the most practical diagnostic modality, demonstrating a dilated esophagus with distal tapering (“bird-beak” appearance) and delayed contrast clearance [1-5]. TBE established the diagnosis in all our cases. UGIE aids in excluding secondary causes such as peptic or congenital strictures and eosinophilic esophagitis. None of our patients had features of Allgrove (Triple-A) syndrome, which is characterized by achalasia, alacrimia, and adrenal insufficiency.

Medical therapy with nitrates or calcium channel blockers is rarely used in children because of limited efficacy data. Botulinum toxin injection may provide transient relief in older children but requires repeated sessions [3]. Peroral endoscopic myotomy (POEM) is widely utilized in adults and older children; however, its safety, efficacy, and feasibility have not been evaluated in infants [6]. Surgical Heller’s myotomy remains the definitive treatment but is technically challenging at this age [7].

In our series, three of four infants (75%) achieved clinical remission without complications and remained symptom-free at a median follow-up duration of 4.5 months. Previous pediatric studies have reported variable outcomes with pneumatic dilation and Heller’s myotomy. Hamza et al. observed durable symptom relief with Heller’s myotomy but noted procedural challenges in smaller children [4]. Babu et al. reported good short-term results with pneumatic dilation in older children while highlighting the risk of perforation and balloon-size limitations [5]. In contrast, our experience with endoscopic CRE balloon dilatation in infants showed comparable short-term efficacy without complications, underscoring its feasibility in this younger age group. Similar favorable outcomes have been described in infant case reports from India [6,7]. These observations suggest that endoscopic CRE dilatation is a reasonable minimally invasive option for infantile achalasia, particularly in settings where surgical expertise or resources are limited.

## Conclusions

Achalasia cardia should be suspected in infants presenting with recurrent vomiting, feeding difficulties, aspiration, and poor weight gain that is refractory to gastroesophageal reflux disease (GERD) therapy. Although congenital esophageal stenosis is an important differential diagnosis in this age group, careful radiologic and endoscopic evaluation can help distinguish between the two conditions. Endoscopic controlled radial expansion (CRE) balloon dilatation appears to be a safe, effective, and minimally invasive treatment option for symptom relief in infantile achalasia. Sustained nutritional improvement at follow-up supports its feasibility and potential as an early therapeutic strategy. Nonetheless, this experience underscores the safety and feasibility of the approach in early infancy and warrants further prospective studies with larger cohorts and longer follow-up to establish its long-term outcomes.
